# Mechanical performance under dynamic loading of rubberized asphalt mixtures with highly-porous vesicular aggregate

**DOI:** 10.1038/s41598-022-24197-3

**Published:** 2022-11-19

**Authors:** Miguel A. Franesqui, Jorge Yepes, Juan Gallego

**Affiliations:** 1 Grupo de Fabricación Integrada y Avanzada-Departamento de Ingeniería Civil, Universidad de Las Palmas de Gran Canaria (ULPGC), Campus de Tafira, 35017 Las Palmas de Gran Canaria, Spain; 2 Departamento de Ingeniería Civil-IOCAG, Universidad de Las Palmas de Gran Canaria (ULPGC), Campus de Tafira, 35017 Las Palmas de Gran Canaria, Spain; 3 Grupo de Investigación en Ingeniería de Carreteras, Departamento de Ingeniería del Transporte, Urbanismo y Territorio, Universidad Politécnica de Madrid (UPM), c/Profesor Aranguren s/n, 28040 Madrid, Spain

**Keywords:** Engineering, Civil engineering

## Abstract

In volcanic regions, the use of certain abundant aggregates of scoriaceous nature with high porosity to manufacture bituminous paving mixtures is a major problem due to the excessive heterogeneity, absorption and limited strength of these aggregates. Consequently, the properties of the mixtures often do not meet technical specifications. The aim of this research is to study the improvement of the mechanical performance of asphalt mixtures with these residual volcanic aggregates by using binders modified with rubber from end-of-life tyres, since environmental and economic requirements make it necessary to reuse both types of waste. Laboratory studies determining the compactability, dynamic stiffness, fatigue resistance and elastic constants have made it possible to characterise the mechanical performance of these mixtures during production and in service, and to compare them with conventional mixtures. It was found that the use of tyre rubber modified bitumen makes compaction somewhat more difficult, but reduces the particle size degradation of the porous aggregates and improves the mixture performance and durability, showing higher stiffness moduli and increased resistance to fatigue.

## Introduction

The mineral aggregate is, in quantitative terms, the main constituent of any asphalt mixture (approx. 92–96% of the total weight and approx. 81–87% of the volume, according to most specifications of bituminous mixtures for paving. (See for example the European Standards EN 13108^1^). This mineral skeleton provides the friction component, which contributes to the resistance of the mixture, depending on the size, shape, angularity, gradation, surface roughness, porosity, intergranular contacts, cleanliness and mineral composition of aggregate particles. Highly-porous vesicular and scoriaceous aggregates are the most frequent lithotypes in volcanic regions, consisting of approximately 75% of the rock mass that can be used and capitalized^2^. Nevertheless, according to previous studies^3^, their excessive heterogeneity and variability, superior absorption and consumption of binder and energy to evaporate water—due to the alveolar structure—, and non-cubic shape, make these aggregates marginal and inadequate for structural materials according to standard technical specifications. Thus, an important part of the production is rejected at the quarries. On the contrary, these volcanic lightweight aggregates present some other interesting properties such as low thermal conductivity, freeze–thaw resistance and low density^4^.

Most previous studies regarding bituminous mixtures with volcanic aggregates compared the stripping, creep behaviour and rutting properties of the mixtures with basalt and with calcareous aggregates or they have combined different proportions of basalt and limestone^5–7^. Notwithstanding, there are relatively few works regarding the sole use of marginal volcanic aggregates in asphalt mixtures^8–10^ and none of them have reported the dynamic performance nor the production and workability characteristics of asphalt mixtures with vesicular and scoriaceous volcanic aggregates.

The waste from used tyres is also an important environmental problem in volcanic territories with highly protected natural areas and therefore reuse on-site is essential. The recycling of crumb rubber from reclaimed end-of-life tyres (ELT) used as a modifier of the asphalt binder (crumb rubber modifier or CRM) for asphalt mixtures is an interesting alternative. Rubberized asphalt (RA), or “wet process”, is a blend of asphalt binder and ground recycled tyre rubber powder from scrap ELT. The rubber particles react in the hot asphalt and cause the rubber swelling. However, reuse and recycling of waste granulated rubber is a challenging problem owing to its three-dimensional cross-linked structure and the rubber devulcanization may offer a solution^11^. The blending of asphalt with rubber at high temperature promotes this process.

Volumetric properties are crucial in the mix-design process and performance of bituminous mixtures. Excessive air void content (mixture porosity) may be the main origin of lower fracture toughness and reduced fatigue life^12,13^. In addition, it can induce age hardening and therefore pavement cracking^14^. On the contrary, asphalt mixtures with very low air void content can cause extreme plastic deformations by traffic loading and the binder´s thermal expansion at high temperature. Nevertheless, the analysis of production, workability and compactability characteristics of RA mixtures with vesicular volcanic aggregates has not been tackled before. Furthermore, the coarse fractions of aggregates employed in bituminous mixtures for pavements require not only satisfactory resistance to surface abrasion under traffic but also sufficient resistance to crushing under compaction machines during construction. This could be more critical with highly-porous volcanic aggregates, but studies regarding particle fragmentation due to compaction energy and the possible benefit of using rubberized binders to reduce this effect have not been performed until now either.

Fatigue performance and stiffness modulus are the principal dynamic mechanical characteristics of a bituminous mixture. Fatigue is one of the most important failure modes in asphalt pavements due to repeated heavy traffic loads (load-related fatigue cracking). The main deterioration mechanisms of flexible pavements are due to a cumulative process of incremental structural damage, produced by strains or by stresses at certain critical points of the pavement structure, which is accumulated throughout its service life. When this incremental damage reaches the maximum value (cumulative damage factor = 100%), the pavement structure is said to have reached structural failure^15^. The improvement of fatigue life using CRM is therefore of great interest in pavement engineering in order to extend the life cycle of pavements. Previous studies have shown that RA seems to enhance the fatigue resistance^16,17^, thanks to the improved rheological properties of the rubberized binders, enabling the production of paving materials with extended in-service life.

On the other hand, the resilient modulus is one of the crucial properties in the analytical design of pavement structures in order to prevent permanent deformations (rutting), to calculate the optimum thickness for each layer, and it is also a decisive parameter used for characterizing the resistance to failure and evaluating the material quality. A high stiffness modulus allows pavement structures with thinner asphalt layers (increases their bearing and stress distribution capacities) and sufficient resistance to rutting. However, the stiffness of asphalt mixtures must not be excessive in order to guarantee adequate ductile and flexible behaviour and thus avoid fatigue cracking at intermediate temperatures or brittle cracking at low temperatures. The addition of crumb rubber to asphalt mixtures generally increases stiffness^18^, as long as this addition does not involve a reduction in the total amount of binder material.

The effect of the rubberized binders on the stiffness and the fatigue performance of the mixtures also depends on the quality of the aggregates used. With high-quality natural aggregates, studies show that rubberized binders improve the fracture resistance^19^ but it is similar or slightly worse with recycled concrete aggregates for lower initial micro-strains^20^. However, no study on the dynamic performance of the RA mixtures with marginal high-porosity volcanic aggregates has been reported.

In brief, the main limitations of the knowledge concerning these issues are:

- There are very few studies involving marginal porous volcanic aggregates and their possible applications in asphalt mixtures, and these materials are not included in most of standard technical specifications. In consequence, despite the quantitative relevance in many regions, an important part of the production is rejected at the quarries. However, in volcanic areas the use of these local aggregates is an environmental, logistic and economic necessity.
- There are no previous studies regarding the dynamic mechanical performance, nor the workability with different compaction energies, of RA mixtures with highly-porous vesicular and scoriaceous aggregates. But the effect of the rubberized binders on these dynamic properties also depends on the quality of the aggregate.
- With vesiculated aggregates, their high porosity could contribute to changes in the gradation during compaction of asphalt mixtures owing to breakage of particles, with significant increment of the fine fraction. The use of rubberized binders could reduce this negative effect, but this has not been studied previously.

The study at hand focuses on the utilization of the elastomeric waste from ELT with the aim of modifying asphalt bitumen and thus enhancing the dynamic performance of mixtures with highly-vesiculated volcanic aggregates. Hence, the main objective is to reuse the two waste products, reduce negative effects on the environment as well as increase pavement durability. In addition, CRM may reduce the need for the higher amount of asphalt bitumen involved when using high porosity aggregates and even contribute to avoid the fracture of aggregate particles during compaction due to the elastic cushioning effect of the rubber.

Thus, to validate the feasibility of these materials, two studies were conducted: (a) analysis of the mixture production and workability, studying the influence of the compaction energy on the volumetric properties, the compactability, and the gradation change; (b) characterization of the stiffness modulus, fatigue laws, and elastic constants. All the mixtures were produced with crumb rubber modified bitumen (RA mixtures) and volcanic aggregates from high-porosity vesicular basalt of scoriaceous nature. Results were also compared to the mixtures produced with conventional bitumen and the same volcanic aggregates. These properties are fundamental to ensure optimal production, performance and durability regarding asphalt pavements.

## Materials

283 cylindrical specimens, 38 non-compacted samples (used for theoretical maximum density tests) and 24 prismatic specimens of semi-dense asphalt concrete were made in the laboratory with bitumen contents between 5.0% and 7.0% (referred to the total weight of the mixture). According to the European Standard EN 13108-1^1^, this type of mixture is named AC16 surf S (where AC means an asphalt concrete, 16 mm is the maximum aggregate size, “surf” indicates its application on surface layers and S is an abbreviation for semi-dense). The Spanish technical specifications for road works (PG-3)^21^ were followed to produce this type of hot mix asphalt (HMA) and it is in accordance with EN 13108-1. This is a bituminous mixture used extensively for surface courses of different roads, traffic types and climates because it provides a better surface macrotexture, lower deformation (due to its mineral skeleton and void content) and is cheaper to produce compared to dense asphalt concrete.

To ensure consistency, the aggregate fractions used in the mixtures (0–4, 4–10 and 10–20 mm) were mechanically crushed from the same type of volcanic rock in the same quarry (Gran Canaria, Canary Islands, Spain): a highly porous olivinic-piroxenic grey basalt (B-V). Geochemically speaking, it may be considered a basanite with brown anfibol, augite, fenocrystals of olivine and plagioclase. The chemical analysis provided the following composition: SiO_2_ (37.50%); CaO (13.38%); MgO (12.24%); Al_2_O_3_ (9.80%); FeO (9.60%); TiO_2_ (5.40%); Fe_2_O_3_ (4.46%); Na_2_O (2.83%); P_2_O_5_ (1.52%); K_2_O (0.48%); MnO (0.17%); H_2_O (1.67%).

**Figure 1:**
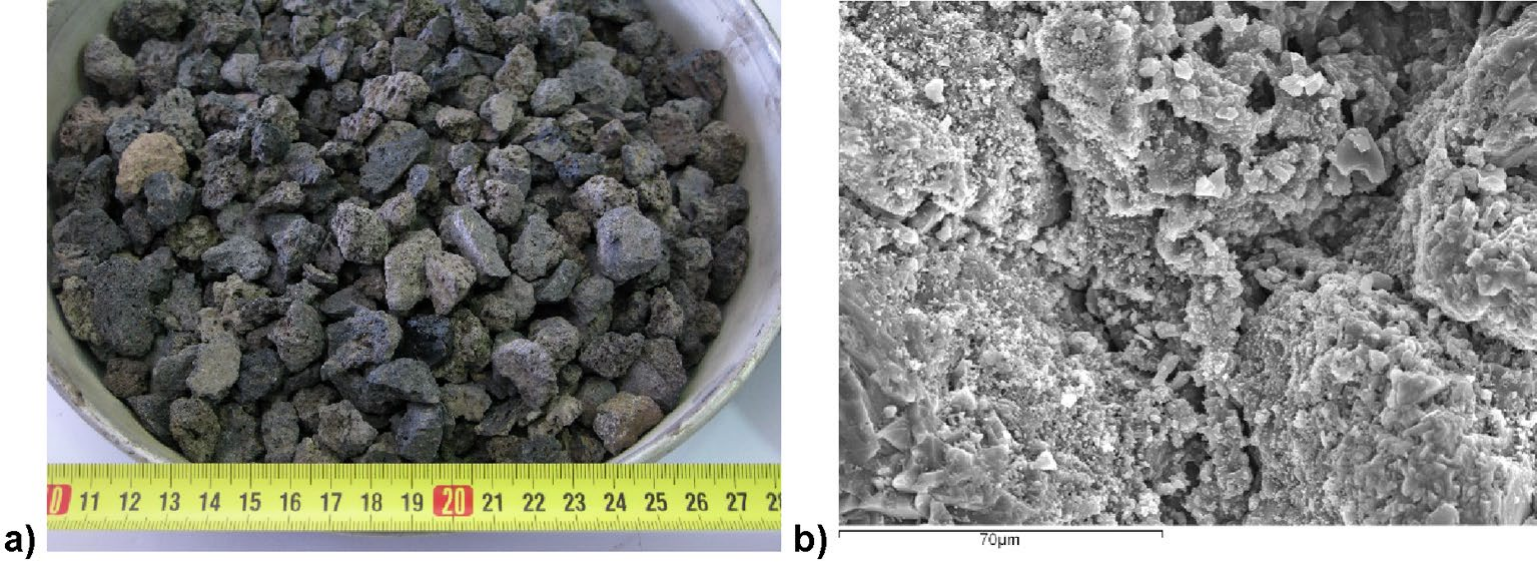
Detail of the B-V volcanic aggregate: (**a**) coarsest fractions (10–20 and 4–10 mm) obtained by mechanical crushing; (**b**) microstructure of this highly-vesiculated basalt (Scanning Electron Microscopy, magnification ×800).

Highly-vesiculated basalt with alveolar and scoriaceous structure constitutes a very common and abundant lithotype of volcanic rock (see Fig. S1 in Supplementary Information). A detail of the aggregate obtained from this rock (B-V) is shown in Fig. 1. For brevity, the main properties are summarized in Table S1 in Supplementary Information. This aggregate offers high water absorption (WA_24_), especially in the finest fraction. Some properties complied with the Spanish specifications for road pavements: polished stone value (PSV), sand equivalent of the fraction 0–4 mm (SE_4_) and flakiness index (FI). Nevertheless, this volcanic aggregate is regarded to be marginal because of the high proportion of non-prismatic particles, and the values of resistance to fragmentation (Los-Angeles coefficient, LA) and to wear (Micro-Deval coefficient, M_DE_). As shown in Table S1 in Supplementary Information, the finest fractions displayed lower particle densities and superior water absorption. WA_24_ of the fraction 0–4 mm (15.5%) was almost three times higher than the 10–20 mm.

As mineral filler (# < 0.063 mm), it was chosen a Portland cement with pozzolanic addition (CEM II/B-P 32.5 R, according to European Norm 197-1), since this cement is commonly produced in volcanic territories where natural pozzolans are abundant.

The reference mixtures used a commercial paving grade bitumen of penetration range 35/50 (penetration with a standardized needle, expressed in tenths of a millimetre, at 25 °C, under a weight of 100 g, during 5 s, according to European Standard EN 1426). The binder of the mixtures with rubber was a rubberized bitumen with the same penetration grade (CRMB 35/50). This was made in the laboratory by adding the crumb rubber modifier (CRM), and is described in section “Methods”. This CRM was previously manufactured using a mechanical grinder at room temperature (50% ELT from cars; 50% ELT from trucks). Only one batch of CRM was used to guarantee consistency. The composition of CRM (thermogravimetric analysis) is: 57.41% polymeric rubber, 32.22% carbon black, 6.02% ash and 4.67% plasticizer and additives. For brevity, the CRM gradation is shown in Table S2 and the main properties of both binders are included in Table S3, both in Supplementary Information.

## Methods

Two types of mixtures were produced in the laboratory in order to compare the effect of the rubberized binder on the compaction properties and on the dynamic mechanical performance of this semi-dense asphalt concrete with porous volcanic aggregate:

- In a first phase, the reference mixtures (AC16 35/50 surf S) with different percentages of conventional bitumen (35/50 pen) were produced and tested.
- In a second phase, the RA mixtures (AC16 CRMB 35/50 surf S) were studied. These were produced with the same aggregate and exactly the same particle size distribution (Fig. S2 in Supplementary Information), the same binder percentages but using the rubberized binder of a similar consistence (described in Table S3 in Supplementary Information), which was produced previously in the laboratory by blending CRM and a base bitumen 50/70 pen (penetration at 25 °C, 100 g, 5 s: 58 × 10^–1^ mm; softening point: 48.6 °C). A bitumen CRMB 35/50 was obtained from the bitumen 50/70, because the elastomer increased the viscosity and consistency of the resulting binder (reduced penetration by 34.5% and increased softening point by 32.1%).

The following laboratory equipment was used to produce the rubberized binder, mixing CRM with hot bitumen: disperser unit (IKA Ultra-Turrax T50 digital, with a propeller agitator, 600–15,000 rpm, max. viscosity 5000 mPas); oil bath (max. 225 °C, with temperature probe, stability and accuracy ± 1.0 °C); and one-litre metal container.

To manufacture the CRMB 35/50, each 50/70 bitumen sample of 600 g was heated to 180 ± 1 °C and then 10% (by weight) of CRM was included in the blending unit with an oil bath and mixed for 60 min at 4000 rpm and 180 °C. In this way, the ultraviolet inhibitors, anti-oxidants and other chemicals present in the rubber shift to the asphalt, together with the elastomeric properties. The result was a reacted rubberized binder of higher consistency.

**Figure 2:**
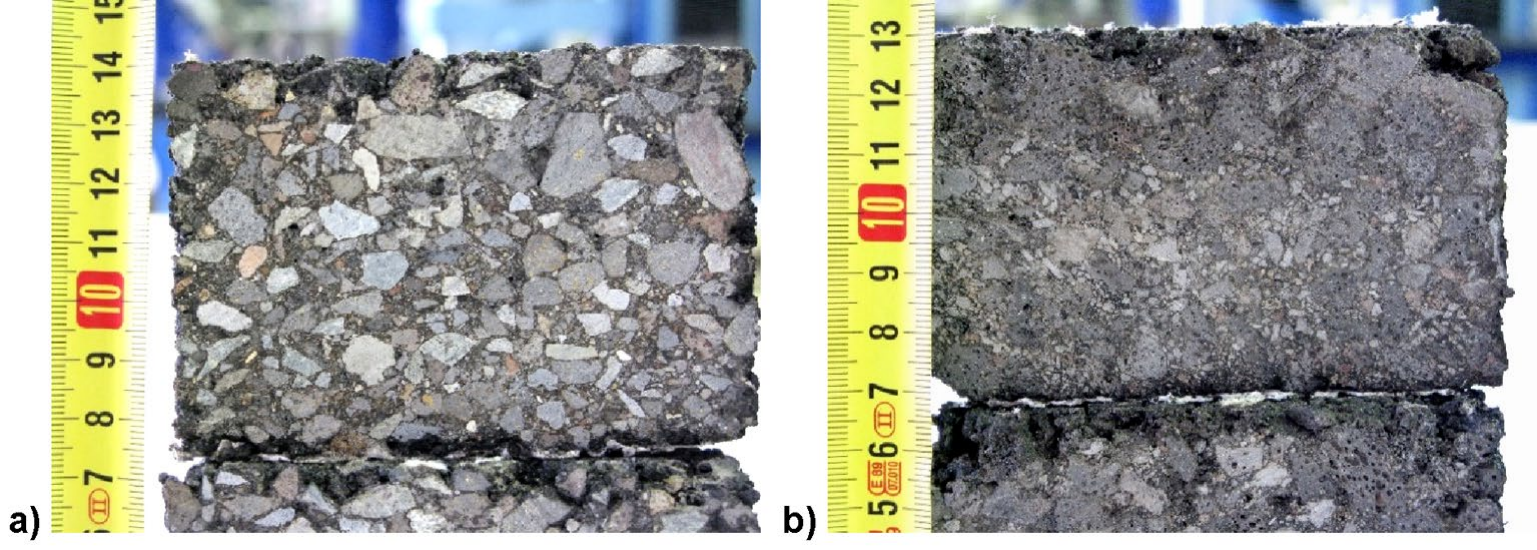
Cut section of cylindrical specimens: (**a**) Ref. mixture made with 6% of bitumen 35/50 pen (by total weight of mixture); (**b**) RA mixture with the same content of rubberized binder (CRMB 35/50).

The reference mixtures were manufactured by heating aggregates and bitumen 35/50 to 170 ± 1 °C. Aggregates were heated during 8 h and bitumen during 3 h in an oven. Finally, they were mixed with one-minute coating by hand and then 2 min in the mixer. The final mixing temperature was 170 ± 1 °C and compaction temperature 160 ± 1 °C. In the RA mixtures the heating temperature of aggregates and bitumen (CRMB 35/50) was 180 ± 1 °C, and they were kept in the mixer unit for 3 min, due to the greater viscosity of the rubber. The final temperature in the mixer was 180 ± 1 °C and the compaction was at 170 ± 1 °C. The cylindrical specimens of diameter 101.6 mm and height 63.5 mm (Fig. 2) were compacted by impact with a Marshall hammer (EN 12697-30) and different energies (50, 75 or 100 blows per side, depending on the laboratory test). The prismatic specimens for fatigue tests were produced from slab specimens of 300 × 300 × 60 mm, compacted by rolling in accordance with EN 12697-33.

The compacted specimens and non-compacted samples received up to three series of characterization tests for each mixture type and bitumen content, with a minimum between three and six specimens for each test. Summarizing, a total of 345 laboratory specimens and samples were tested, with a total number of 65 characterization tests and 480 ultrasound tests on the reference mixtures (without rubber) and 75 and 480 on the RA mixtures, respectively. When required, the test samples were conditioned in a heater-refrigerator during the time to reach the normalized temperature according to standards and maintained during the test if necessary.

The different characterization tests were classified into the following categories:

- Volumetric properties: theoretical maximum density (according to EN 12697-5, Procedure A: volumetric); bulk density (EN 12697-6, Procedure B: saturated surface dry, and Procedure D: geometric); and void characteristics (EN 12697-8) on cylindrical specimens compacted by impact with different energies (number of blows) using the Marshall hammer.
- Compaction properties: compactability test (EN 12697-10) on cylindrical specimens compacted by impact with 2 × 100 blows (EN 12697-30) using the Marshall compactor, monitoring and recording the change of the specimen thickness during the compaction process. Additionally, the gradation change after compaction was determined by sieve analysis of aggregates.
- Dynamic mechanical performance: dynamic stiffness modulus (EN 12697-26, by indirect tensile test on cylindrical specimens [IT-CY] compacted by impact with 2 × 75 blows, k = 0.6, T = 20 °C, f = 2.2 Hz); elastic constants by determining the velocity of ultrasonic pulses (elasticity moduli and Poisson’s ratio at 20 °C, according to EN-12504–4 and BS 1881: Part 203); and resistance to fatigue (EN 12697–24, by four-point bending test on prismatic specimens [4 PB-PR] with 10^6^ cycles, at 20 ºC, 10 Hz).

As for the compactability test using the Marshall compactor, according to the European Standard EN 12697-10 and the method of same sample for all energy levels, the Equation (1) shown in Table S4 in Supplementary Information was used to relate the thickness variation of the specimen, the compaction energy applied and the resistance to compaction.

To obtain the stiffness modulus according to the equation proposed by EN 12697-26 (Equation (2), see Table S4 in Supplementary Information), in each IT-CY test five haversine repeated loading pulses with the corresponding intermediate rest periods were applied, controlling the loading time during tests. The ratio of rest periods to loading time (R/D) were between 20.5 and 22.2. According to Ref.^22^, R/D should be equal or greater to 9 to achieve an acceptable range of error in measurement of resilient modulus. The load surface factor (k, related to the shape of the loading pulse curve or waveform), rise time (from zero load up to peak load), strain and stiffness modulus in each loading pulse were also obtained. Equation (2) provides the dynamic stiffness modulus. According to EN 12697-26, it was verified that the rise time was between 120 and 128 ms, the load surface factor (k) between 0.5 and 0.8, and the strain between 3 and 20 µm. The stiffness modulus measured was corrected by Equation (3) when k ≠ 0.6.

Moreover, as this is a non-destructive test, each specimen was tested at two different diametrically opposite positions. The load amplitude was 2700 N, the pulse frequency 2.2 Hz and the resting time 2750 ms. The temperature was maintained at 20 °C throughout the test. Afterwards, averages of these moduli were calculated and also the mean values for each set of the three specimens with the same bitumen content.

Regarding the fatigue tests and according to European Standard EN 12697-24, in each 4 PB-PR test a repeated haversine load was applied on prismatic specimens. The four supporting points enable a flexural load with a constant strain within the two intermediate clamps of the beam specimen. 12 prismatic beams, obtained by cutting slab specimens, for each type of asphalt mixture were tested at 20 °C. A strain control procedure was used. The levels of micro-strains were chosen, testing different series of specimens, so that a sufficiently wide range of the number of cycles to fatigue (N) could be achieved. In this research micro-strain ranged between 41 and 94 µm. The level of strain for 10^6^ cycles was 50.4 µm for the RA mixture tests.

According to EN 12697-24, the initial stiffness modulus calculated for each mixture specimen (S_mix_) is obtained from the load, displacement and phase angle after 100 load applications. The test continues until the modulus diminishes to half of its initial value or up to the specimen failure. The fatigue law for fatigue life prediction was obtained by linear regression of the logarithms of the number of cycles and the logarithms of the initial strain amplitude, according to Equation (4).

The elastic constants of the asphalt mixtures were determined by measuring the velocity of ultrasonic pulses (EN 12504-4) on the same cylindrical specimens before the stiffness modulus tests, since the ultrasonic test are non-destructive. These tests were performed using a direct transmission scheme, placing both couplant plate contact (CPC) piezoelectric transducers on the middle point of the bases of each cylindrical specimen. Transducers of three different low frequencies were used (24, 54 and 250 kHz), in order to avoid scattering and dispersion of waves by the aggregate particles and pores. Velocities of ultrasonic P-waves (longitudinal compression waves) and S-waves (transverse shear waves) were registered at a constant temperature of 20 °C, as moduli of bituminous materials are temperature-dependent. According to BS 1881: Part 203 and ASTM D2845-00, the ultrasonic elastic constants of the mixtures can be obtained as shown in Equations (5)–(7), assuming an elastic medium.

## Results and discussion

### Mixture production and compactability

- Influence of the compaction energy on the volumetric properties:

Volumetric properties are normally the first characteristics studied in the design of a bituminous mixture because these properties are crucial in its formulation and performance. Inadequate air void content and insufficient density may cause an impoverished mechanical performance^23^.

Theoretical maximum density depends on the aggregate type and gradation as well as on the bitumen type and content. The maximum densities of the rubberized asphalt (RA) mixtures were effectively higher compared to the reference mixtures for the same binder contents (1–3% superior), decreasing in both mixtures proportionally as the bitumen percentage increases due to the lower density of bitumen in comparison with aggregates.

**Figure 3:**
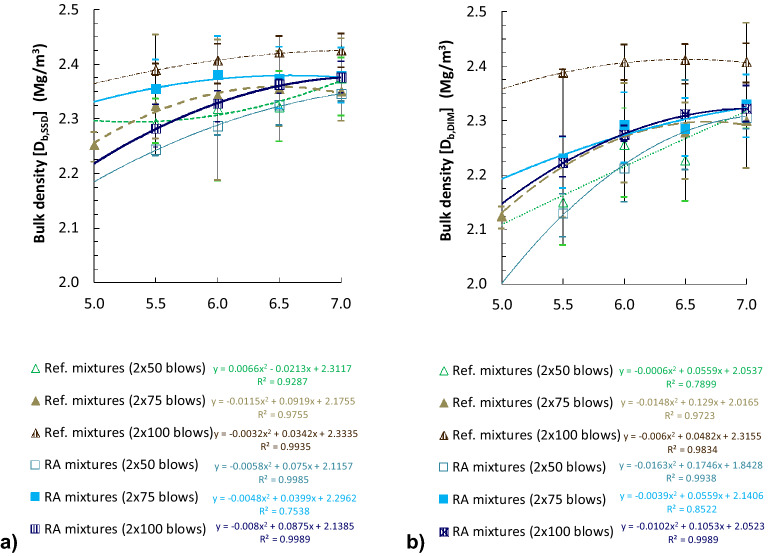
Densities of the bituminous mixtures on cylindrical specimens with different compaction energies: (**a**) bulk density (Proced. B: saturated surface dry, EN 12697-6); (**b**) Bulk density (Proced. D: geometric, EN 12697-6).

Figure 3 represents bulk densities of cylindrical specimens compacted by impact with different energies (blows/side). As shown in this figure, bulk densities of the RA mixtures are similar (differences between 0.2 and 2.2% by saturated surface dry [SSD] procedure; 0.3–2.6% by geometric [DIM] procedure) to the reference mixtures with conventional bitumen, for specimens compacted with 50 and 75 blows/side (with this last compaction energy they are even slightly superior). By contrast, the samples of RA mixtures with 100 blows/side offered an apparently lower density (2.1–4.7% by SSD procedure; 3.7–7.4% by geometric procedure) compared to the reference mixtures. This contrary-to-expected behaviour suggests that the elastic properties of the rubber could cushion the highest dynamic compaction energies (2 × 100 blows), obtaining similar or even lower densities than with 2 × 75 blows. The highest bulk densities were obtained with similar binder contents in both types of mixtures (6.5–7% by total wt. of mix), showing the great binder absorption of these porous volcanic aggregates.

Table 1 outlines the averages of the statistical parameters regarding the results of the different properties. Bulk densities attained for both types of mixtures proved to be homogeneous, particularly in case of RA mixtures (Coefficient of variation: Cv ≤ 1.7% for RA mixtures; Cv ≤ 3.0% for Ref. mixtures).

**Table 1:** Statistical dispersion parameters of the laboratory results.

Property of the mixture	Ref. mixtures^a^		RA mixtures^b^	
	Sd^c^	Cv (%)^d^	Sd^c^	Cv (%)^d^
D_m,V_^e^	100 kg/m^3^	3.98	30 kg/m^3^	0.99
D_b,SSD_^f^ (2 × 75 blows)	60 kg/m^3^	2.74	40 kg/m^3^	1.71
D_b,DIM_^g^ (2 × 75 blows)	70 kg/m^3^	3.03	30 kg/m^3^	1.45
D_b,DIM_^g^ (2 × 100 blows)	30 kg/m^3^	1.18	20 kg/m^3^	1.06
T^h^	7.04 blows	14.15	13.08 blows	32.51
Sm_[IT-CY]_^i^	375.34 MPa	5.98	521.69 MPa	8.48

^a^ Reference mixtures without rubber;^b^ Rubberized asphalt mixtures;^c^ Standard deviation;^d^ Coefficient of variation;^e^ Theoretical maximum density;^f^ Bulk density (saturated surface dry [SSD] procedure);^g^ Bulk density (geometric [DIM] procedure);^h^ Resistance to compaction by impact;^i^ Stiffness modulus by indirect tensile tests on cylindrical specimens.

**Figure 4:**
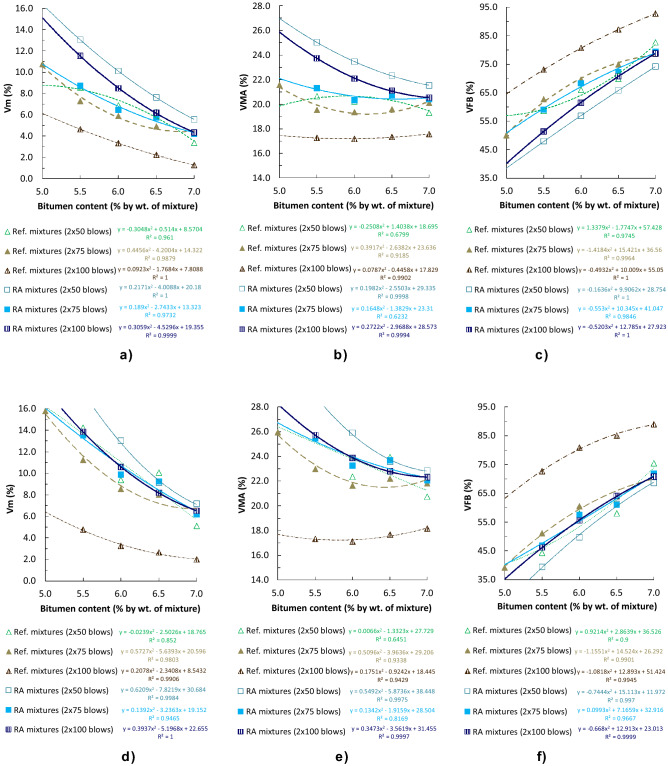
Void characteristics of cylindrical specimens with different compaction energies (EN 12697-8) [(**a**)–(**c**) obtained by SSD bulk density; (**d**)–(**f**) obtained by geometric bulk density].

The air void content (V_m_) and voids in mineral aggregate (VMA) in the RA mixtures were superior compared to the reference mixtures for the same compaction energy (Fig. 4). This result is due to the increased theoretical maximum densities of the RA mixtures. Moreover, the superior air void difference observed when mixtures were compacted with the highest energy (2 × 100 blows) is related to the lower bulk density of RA mixtures in this case, as aforementioned.

A demanding compaction, because of the higher viscosity of the rubberized binder, was also suggested by the superior porosity of the RA mixtures, even though they were manufactured at a temperature 10 ºC superior. This was also noted during laboratory production of RA mixtures. In Ref.^24^ it was proved that the temperature affects directly the volumetric characteristics of RA mixtures. In Ref.^25^ comparable results were achieved in the case of recycled concrete aggregates. The difference in V_m_ was minimum in compacted specimens by 2 × 75 blows (0.1–1.4%, by SSD procedure; 0.2–2.3% by geometric procedure), moderate for 2 × 50 blows (1.4–4.5%; 0.9–3.6%, respectively) and maximum for 2 × 100 blows (3.1–6.9%; 4.5–9.1%, respectively). This proves that the elastomeric character of the rubber prevents the highest dynamic compaction energies to be effective, producing inferior densities in the compacted specimens than some with less energy. Figure 4c and f show the percentage of voids filled with bitumen (VFB). In the case of RA mixtures, the percentage was lower.

**Figure 5:**
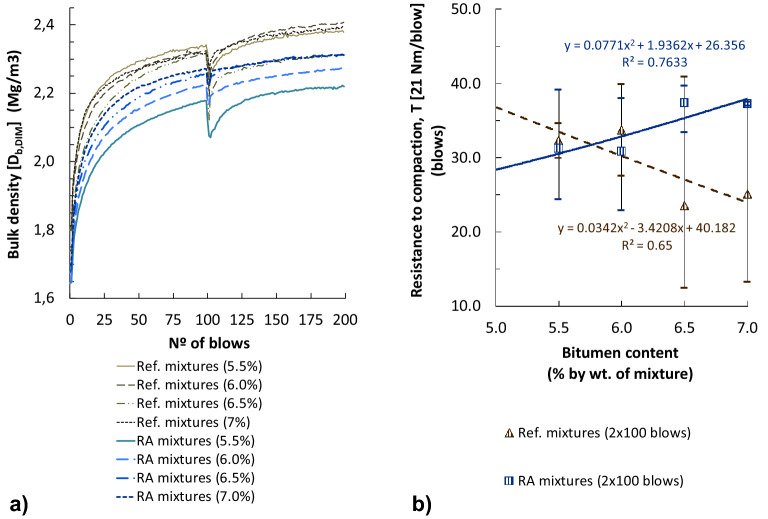
Compactability test results on cylindrical specimens (EN 12697-10): (**a**) bulk density (Proced. D: geometric) for different bitumen contents; (**b**) resistance to compaction of specimens compacted with 2 × 100 blows.

(b) Compactability tests:

Compactability test results by the method of same sample for all energy levels (EN 12697-10) are shown in Fig. 5. The geometric bulk density of RA mixture specimens compacted by the Marshall impact compactor with the maximum energy (100 blows per side) resulted inferior to the reference mixtures, as also previously shown in Fig. 3b. This occurred with all the bitumen contents studied, though RA mixtures were compacted at a temperature 10 °C higher.

Figure 5b shows the resistance to compaction by impact (T) of cylindrical specimens up to 2 × 100 blows (calculated according to the procedure suggested by EN 12697-10). With the conventional bitumen (Ref. mixtures), such resistance to compaction is reduced as the bitumen percentage increased, showing that with an insufficient binder content the internal friction among these highly-porous and rough particles is not overcome. However, with the RA mixtures the resistance to compaction by impact was in general higher than with conventional bitumen and increased with the bitumen content, due to the higher viscosity of the rubberized binder.

(iii) Gradation change due to compaction:

Excessive fragmentation of particles due to compaction energy could change the particle size distribution of the mineral skeleton increasing the percentage of fine particles and thus, modify the internal structure of the asphalt mixture and its volumetric and mechanical properties. Some studies tested against impact these type of volcanic aggregate particles and evaluated their resistance to break-up, and found a dependence of aggregate stability on primary particle size distribution^26^. Our work also aims to verify if the elastomeric characteristics of the rubberized binders maybe could reduce this negative consequence. Previous research with rubberized cement concrete has shown increased impact resistance, even under low temperatures, due to the rubber particles^27^.

**Figure 6:**
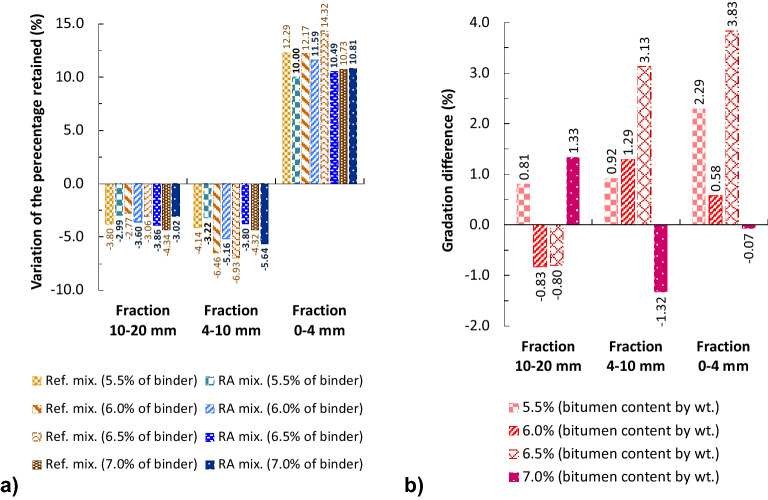
Aggregate gradation change of cylindrical specimens after compaction: (**a**) Variation of the percentage retained for each aggregate fraction (negative values indicate reduction of the fraction); (**b**) gradation difference between RA mixtures and Ref. mixtures (positive values indicate lower gradation change by employing the rubberized binder).

The effect of the rubberized binder on highly-porous aggregate gradation change following compaction was carried out by comparing the particle size distribution before and after impact compaction on the cylindrical specimens with the maximum number of blows (2 × 100) using the Marshall compactor. The variation of the percentage retained between sieves for each aggregate fraction was determined (Fig. 6a) and subsequently the differences between the RA mixtures and the reference mixtures (Fig. 6b).

As Fig. 6a shows, an increase of the fine fraction (0–4 mm) took place in both mixtures as a consequence of the breaking of coarser particles (reduction in sizes 10–20 and 4–10 mm). However, the grain size degradation was slightly lower in the RA mixtures (the loss of the 10–20 mm fraction was 0.8–1.3% less, depending on the bitumen content; as for the 4–10 mm it was 0.9–3.1% less; the 0–4 mm fine fraction increment was 0.6–3.8% lower). Generally speaking, the particle size modification is lower as the rubberized binder percentage is increased which again suggests a cushioning effect of the highest dynamic energies owing to the rubber incorporated in the binder, increasing its elastic properties, and the higher viscosity of the mixtures with CRM bitumen.

### Dynamic stiffness modulus

**Figure 7:**
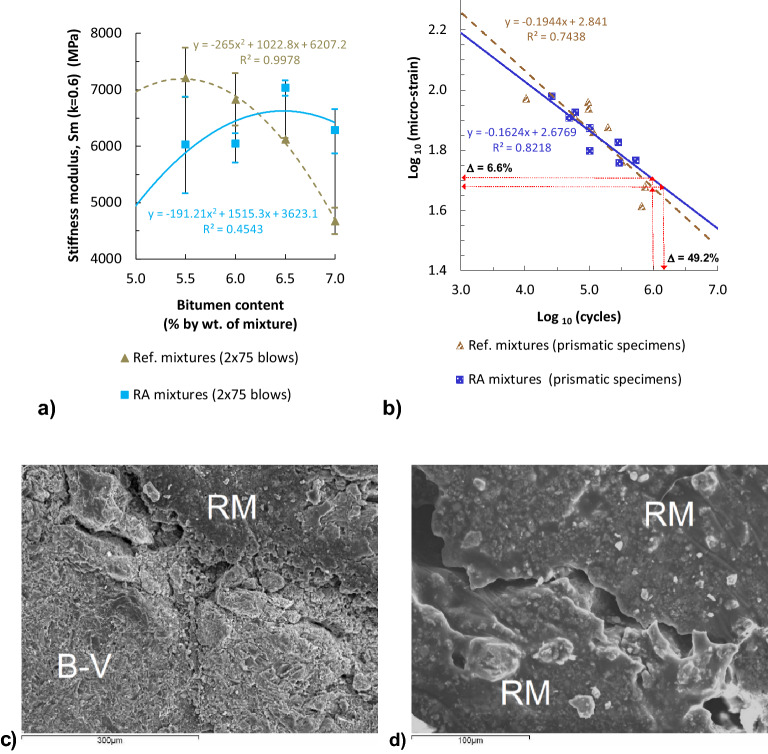
Dynamic mechanical properties: (**a**) stiffness modulus by IT-CY tests (EN 12697-26); (**b**) fatigue laws by 4PB-PR tests (EN 12697-24) with a bitumen content of 6% (by total wt. of mixture); (**c**) fatigue micro-crack at the interfacial contact between the volcanic aggregate particle (B–V) and the mastic of rubberized binder (RM) [magnification ×200]; (**d**) fatigue micro-crack in the RM [magnification ×400].

The stiffness of a material such as asphalt concrete depends on the stiffness of both the cementitious matrix (asphalt mastic) and the aggregate^28^. Dynamic stiffness modulus by indirect tensile tests on cylindrical specimens (IT-CY) [with load surface factor k = 0.6, at 20 °C, f = 2.2 Hz, and compacted with 2 × 75 blows] are shown in Fig. 7a. According to laboratory results, the stiffness modulus of the RA mixtures with bitumen contents of 5.5% and 6.0% was inferior to the reference mixtures (19.7% and 13.0%, respectively). Nonetheless, for higher binder content the dynamic stiffness proved to be greater (14.9% for 6.5% of bitumen and 34.4% for a content of 7%). Therefore, the elastomeric effect of the rubber may be also observed in these results: higher stiffness with higher rubberized binder contents. The maximum stiffness of an RA mixture was obtained with 6.5% of binder, a content 1% superior than with the reference mix.

Some other studies also stated that bituminous mixtures with rubberized binders present higher stiffness modulus^20^, owing to the increased stiffness of the binders modified with tyre rubber powder^29^. However, the results of our study have shown that, when the content of the rubber-modified binder was lower than 6.5% (approx. for contents lower than 6.3%, according to the regression curve), these mixtures showed inferior stiffness compared to the mixtures without rubber. This could probably be explained because under these conditions the vesicular aggregates have more influence on the stiffness modulus, due to their porous structure and weakness. Furthermore, the mixtures have a higher air void volume than the reference mixtures with these lower contents of rubberized binder, owing to the worst coating and absorption by the pores of the rubber-modified binder, which is more viscous, leading to a lower stiffness of the bituminous mixture.

The results present a statistical dispersion with an average coefficient of variation (Cv) below 8.5% for the RA mixtures and 6.0% in case of reference mixtures (Table 1).

### Fatigue laws

Cracking resistance of asphalt mixtures depends on their ductility, extensibility, and tension strength. These properties vary with the content of asphalt binder and with the stiffness of the mixture. The resistance to fatigue under repeated loads of the RA mixture with 6% of binder by four-point bending test on prismatic specimens (4PB-PR, at 20 ºC, 10^6^ cycles, 10 Hz) was slightly superior to the reference mixture with identical binder content, as the fatigue law slope for the former resulted less pronounced (Fig. 7b). Results for the RA mixture also shown a better fitted regression curve (R^2^ = 0.82 for RA mixture; R^2^ = 0.74 for Ref. mixture). Moreover, the RA mixture showed higher fatigue life for initial micro-strains greater than 69.6 µm (intersection of both fatigue laws). The initial micro-strain for an expected service life until failure of 10^6^ cycles increased by 6.6% with respect to the reference mixture without rubber, which represents an increment of the expected number of fatigue cycles of 49.2% to reach the aforementioned micro-strain. Figure 7c and d present scanning electron micrographs, showing fatigue micro-cracks with fine microscopic ridges and well pronounced dimples avoiding smooth fracture surfaces.

### Ultrasound elastic constants

The feasibility of ultrasonic methods in asphalt mixtures has been already demonstrated, despite the characteristic heterogeneity and air void content of these materials. Some studies used direct transmission methods measuring ultrasonic pulse velocity of P-waves (compression waves) and S-waves (shear waves) to obtain the elastic constants of cylindrical specimens^30^. In this work, the feasibility of determining these elastic constants by means of ultrasounds in the case of semi-dense asphalt mixtures with an extremely porous mineral aggregate has been attempted.

**Figure 8:**
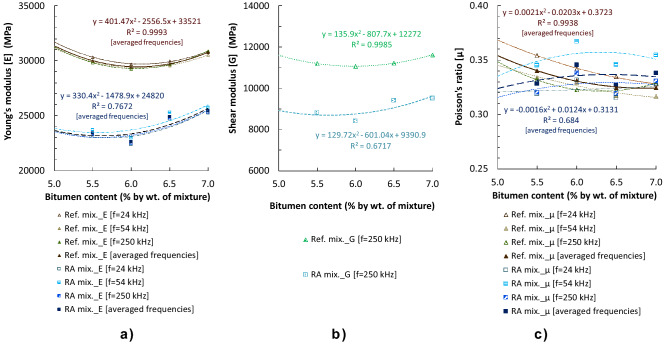
Elastic constants obtained by ultrasounds on cylindrical specimens (compacted by impact with 2 × 75 blows), at 20 °C and different pulse frequencies: (**a**) Young’s modulus (E); (**b**) Shear modulus (G); (**c**) Poisson’s ratio (μ).

Figure 8 compares the elastic constants of the Ref. mixtures and the RA mixtures calculated applying the aforementioned equations (Eq. (5) to Eq. (7) of Table S4 in Supplementary Information) to the results of ultrasound velocities on cylindrical specimens compacted by impact with 2 × 75 blows. The lower elasticity moduli of RA mixtures are due to their higher air void content (Fig. 4). As shown in Fig. 8, there are no differences in results of Young´s modulus (E) with these different low frequencies of pulses (24, 54 and 250 kHz), though results for Poisson´s ratio (μ) shown to be more influenced by the frequency. This ratio varied from to 0.32 to 0.35 for Ref. mixtures and from 0.32 to 0.37 in RA mixtures.

## Conclusions

Considering the results and discussion of this experimental study, the following conclusions may be drawn:

- The exploitation of locally available poor-quality aggregates has become an environmental, logistic, and economic need in many regions where environmental protection is strict or where economic resources are restricted. This research has proved that the use of crumb rubber from end-of-life tyres in asphalt mixtures with marginal volcanic aggregates becomes an efficient manner to reuse both residual materials, as well as improving certain mechanical properties of the mixtures.
- The higher viscosity and elastic properties of the rubberized binder produced a cushioning effect of the compaction energies during the production of these rubberized asphalt (RA) mixtures with vesicular basalt. Consequently, the air void content of the RA mixtures is superior (up to 3–7% higher, compared to mixtures with conventional bitumen at the same compaction energy), and so compaction is more critical and sensitive to the temperature and energy during compaction works.
- The presence of the rubber slightly reduced (0.6–4%) the grain size degradation of the highly porous aggregate during compaction, decreasing the breaking of the coarser particles, thus, maintaining the characteristics of the pavement mineral skeleton and improving durability. This is considered a favourable contribution of the rubberized binder.
- Results show that the elastomeric properties of the rubber increase significantly (15–34%) the dynamic stiffness modulus of the RA mixtures with higher binder contents. This means that it is possible to utilize these RA mixtures with poor-quality aggregates, even with elevated bitumen percentages in structural pavements under heavy traffic loads.
- Such an increase of the stiffness modulus would mean in practice a reduction of the asphalt pavement thickness by 6–13%, or an increase in the estimated service life by 43–90% if maintaining the same thickness, respectively (for a pavement designed for 3.6·10^6^ equivalent-to-130 kN load axles during 20 years of service life).
- The resistance to fatigue under dynamic loads of the RA mixture was superior compared to the mixture with conventional bitumen. The micro-strain needed for an expected service life of 10^6^ cycles increased by 7%; this represents an increment of the expected number of fatigue load cycles of 49% and consequently, a similar increase of the pavement durability.

The study of the potential contribution of the rubberized binders on some different strength properties of these mixtures under other types of actions (e.g. indirect tensile strength tests, flow number tests and fracture resistance under semi-circular bend tests) is proposed for future research.

## Supplementary Information

Supplementary Information.:

## Data Availability

All data generated or analysed during the laboratory tests are included in this published article (and its Supplementary Information files). Raw data registered directly by testing instruments are available from the corresponding author under request.

## References

[1] European Committee for Standardization, CEN. In *Bituminous mixtures. Material specifications. Part 1: Asphalt Concrete; Part 2: Asphalt Concrete for very thin layers; Part 5: Stone Mastic Asphalt; Part 7: Porous Asphalt*. Bruxelles. 2006.

[2] García-González C, Yepes J, Franesqui MA (2020). Geomechanical characterization of volcanic aggregates for paving construction applications and correlation with the rock properties. Transp. Geotech..

[3] Franesqui, M. A., Castelo-Branco, F., Azevedo, M. C. & Moita, P. Construction experiences with volcanic unbound aggregates in road pavements. In *Volcanic Rock Mechanics* (ed. Olalla et al.) 241–247 (London: Taylor & Francis Group, 2010). 10.1201/b10549-36.

[4] Nawaz W (2019). Experimental study on the shear strength of reinforced concrete beams cast with Lava lightweight aggregates. ARC Civ. Mech. Eng..

[5] Al-Khateeb GG, Khedaywi TS, Obaidat TIA, Najib AM (2013). Laboratory study for comparing rutting performance of limestone and basalt superpave asphalt mixtures. J. Mater. Civ. Eng..

[6] Behiry AE (2016). Optimisation of hot mix asphalt performance based on aggregate selection. Int. J. Pav. Eng..

[7] Iskender E (2013). Rutting evaluation of stone mastic asphalt for basalt and basalt-limestone aggregate combinations. Compos. Part B-Eng..

[8] Faustino, R. P., O'Connell, M. J., Valencia, N. R., Ford, W. Making effective use of volcanic ash in road-building in the Philippines. In *Proceedings of the Eastern Asia Society for Transportation Studies, vol. 5* 868–876 (2005) http://citeseerx.ist.psu.edu/viewdoc/download?doi=10.1.1.543.2240&rep=rep1&type=pdf.

[9] Naji JA, Asi IM (2008). Performance evaluation of asphalt concrete mixes containing granular volcanic ash. J. Mater. Civ. Eng..

[10] Akbulut H, Gurer C, Cetin S (2011). Use of volcanic aggregates in asphalt pavement mixes. Proc. Inst. Civ. Eng.-Transp..

[11] Simon DÁ, Pirityi DZ, Bárány T (2020). Devulcanization of ground tire rubber: Microwave and thermomechanical approaches. Sci. Rep..

[12] Aliha MRM, Fazaeli H, Aghajani S, Moghadas Nejad F (2015). Effect of temperature and air void on mixed mode fracture toughness of modified asphalt mixtures. Constr. Build. Mater..

[13] Ma T, Zhang Y, Zhang D, Yan J, Ye Q (2016). Influences by air voids on fatigue life of asphalt mixture based on discrete element method. Constr. Build. Mater..

[14] Svasdisant, T., Schorsch, M., Baladi, G. Y., Pinyosunun, S. (2002) Mechanistic analysis of top-down cracks in asphalt pavements. *Transp Res Record*. **1809**, 126–136. 10.3141/1809-15.

[15] American Association of State Highway and Transport Officials. In *Mechanistic-Empirical Pavement Design Guide. A Manual of Practice, 3rd Edition*. (AASHTO, 2020). ISBN: 978-1-56051-7481. https://store.transportation.org/Item/CollectionDetail?ID=196.

[16] Raad L, Saboundjian S (1998). Fatigue behaviour of rubber modified pavements. Transp. Res. Record..

[17] Wang H, Dang Z, Li L, You Z (2013). Analysis on fatigue crack growth laws for crumb rubber modified (CRM) asphalt mixture. Constr. Build. Mater..

[18] Moreno-Navarro F, Sol-Sánchez M, Rubio-Gámez MC, Segarra-Martínez M (2014). The use of additives for the improvement of the mechanical behaviour of high modulus asphalt mixtures. Constr. Build. Mater..

[19] Mull MA, Stuart K, Yehia A (2002). Fracture resistance characterization of chemically modified crumb rubber asphalt pavement. J. Mater. Sci..

[20] Pasandín AR, Pérez I (2017). Fatigue performance of bituminous mixtures made with recycled concrete aggregates and waste tire rubber. Constr. Build. Mater..

[21] Spanish Ministry of Infrastructures. In *Spanish General Technical Specifications for Roads and Bridges (PG-3, Art. 542 & 543)*. Orden FOM/2523/2014. Madrid, Spain. 2014 (in Spanish).

[22] Ghanizadeh AR, Fakhri M (2013). Effect of waveform, duration and rest period on the resilient modulus of asphalt mixes. Procedia Soc. Behav. Sci..

[23] Rodríguez-Alloza AM, Gallego J (2017). Volumetric characteristics and compactability of asphalt rubber mixtures with organic warm mix asphalt additives. Mater. Constr..

[24] Lee S, Amirkhanian SN, Kwon S (2008). The effects of compaction temperature on CRM mixtures made with the SGC and the Marshall compactor. Constr. Build. Mater..

[25] Pérez I, Pasandín AR (2017). Moisture damage resistance of hot-mix asphalt made with recycled concrete aggregates and crumb rubber. J. Clean Prod..

[26] Mueller SB, Kueppers U, Ametsbichler J (2017). Stability of volcanic ash aggregates and break-up processes. Sci. Rep..

[27] Yu Y, Jin Z, Zhu H, Song H (2021). Effect of rubber particles on impact resistance of concrete at a temperature of −20 ℃. ARC Civ. Mech. Eng..

[28] Revilla-Cuesta V, Skaf M, Santamaría A, Romera JM, Ortega-López V (2022). Elastic stiffness estimation of aggregate–ITZ system of concrete through matrix porosity and volumetric considerations: Explanation and exemplification. ARC Civ. Mech. Eng..

[29] Rodríguez-Alloza AM, Gallego J, Giuliani F (2017). Complex shear modulus and phase angle of crumb rubber modified binders containing organic warm mix asphalt additives. Mater. Struct..

[30] Tigdemir, M., Kayoncuoglu, S. F., Kalyoncuoglu, U. Y. (2004) Application of ultrasonic method in asphalt concrete testing for fatigue life estimation. *NDT&E Int*. **37:** 597–602. 10.1016/j.ndteint.2004.03.006

