# Supplementary material for: Mechanical performance under dynamic loading of rubberized asphalt mixtures with highly-porous vesicular aggregate
Source: Sci Rep. 2022 Nov 19;12:19973. doi: 10.1038/s41598-022-24197-3 (PMC9675735; doi:10.1038/s41598-022-24197-3)
Supplement: Supplementary file 1 — Supplementary Information. [file 41598_2022_24197_MOESM1_ESM.pdf]

Mechanical performance under dynamic loading of rubberized asphalt mixtures with highly-porous vesicular aggregate

Miguel A. Franesqui [1,*], **Jorge Yepes** [2], **Juan Gallego** [3]

[1] Grupo de Fabricación Integrada y Avanzada – Departamento de Ingeniería Civil, Universidad de Las Palmas de Gran Canaria (ULPGC), Campus de Tafira, 35017 Las Palmas de Gran Canaria, Spain.

[2] Departamento de Ingeniería Civil – IOCAG, Universidad de Las Palmas de Gran Canaria (ULPGC), Campus de Tafira, 35017 Las Palmas de Gran Canaria, Spain.

[3] Grupo de Investigación en Ingeniería de Carreteras, Departamento de Ingeniería del Transporte, Urbanismo y Territorio, Universidad Politécnica de Madrid (UPM), c/ Profesor Aranguren s/n, 28040 Madrid, Spain.

[*] Corresponding author. E-mail address: miguel.franesqui@ulpgc.es (M.A. Franesqui).

Supplementary Information

1. Tables

Table S1. Characterization properties of the volcanic aggregate

	**Aggregate fractions**				**Specified values by technical regulations**
	**# 10–20 mm**	**# 4–10 mm**	**# 0–4 mm**	**Mineral filler (# < 0.063 mm)**	
Lithotype	Vesicular and scoriaceous grey basalt (B-V)			100% CEM II/B-P 32.5 R [EN 197-1]	-
% (by wt. of total aggregate)	20.93	36.27	38.88	3.92	-
ρ_a[a] (Mg/m^3)	2.89	2.88	2.45	-	-
ρ_{SSD}[b] (Mg/m^3)	2.63	2.56	2.36	-	-
ρ_{rd}[c] (Mg/m^3)	2.35	2.37	2.23	-	-
WA_{24}[d] (%)	5.8	8.3	15.5	-	≤ 3 – 3.5 % (*); ≤ 6 – 7 % (**)
FI[e]	6	6	-	-	≤ 20 – 30
Cc[f]	56	60	-	-	≥ 70 – 100
SE_4[g]	-	-	73	-	> 55
LA[h]	29	28	-	-	≤ 20 – 25
M_{DE}[i]	17	23	-	-	≤ 16 – 18
PSV[j]	60	60	-	-	≥ 50 – 56

[a]Particle density [apparent] [EN 1097-6]; [b]Particle density [saturated surface dry] [EN 1097-6]; [c]Particle density [dry] [EN 1097-6]; [d]Water absorption of particles after 24 hours [EN 1097-6]; [e]Flakiness index [EN 933-3]; [f]Percentage of particles with more than 50% of their surface crushed or broken [EN 933-5]; [g]Sand equivalent of fraction 0-4 mm [EN 933-8]; [h]Los-Angeles coefficient [EN 1097-2]; [i]Micro-Deval coefficient [EN 1097-1]; [j]Polished stone value [EN 1097-8]; (*)Recommended limits for structural cement concrete; (**)Recommended limits for recycled aggregates.

Table S2. Particle size distribution of reclaimed CRM from used tyres

Particle size [EN 933-2] (mm)	% passing
1	100.0
0.5	94.1
0.25	23.7
0.125	3.7
0.063	0.4

Table S3. Characterization properties of the asphalt binders

		Ref. mixtures[a]	RA mixtures[b]
Bitumen type	Penetration Grade	35/50	CRMB 35/50[g]
	Performance Grade	PG 70-16	PG 76-10
% (by wt. of mixture)		5.0 – 7.0	5.5 – 7.0
Density[c] (Mg/m^3)		1.042	1.028
Pen.[d] ($x10^{-1}$ mm)		44	38
Soft. Point R&B[e] (ºC)		51.6	64.2
Viscosity[f] (cP)	at 60 ºC	51,000	215,000
	at 135 ºC	600	2,100
	at 150 ºC	250	890

[a]Reference mixtures without rubber; [b]Rubberized asphalt mixtures; [c]Bitumen density according to EN 15326; [d]Penetration at 25 ºC, 100 g, 5s [EN 1426]; [e]Softening point by Ring-and-Ball test [EN 1427]; [f]Dynamic viscosity by Brookfield rotational viscometer [EN 13302]; [g]Composition of CRMB 35/50 (by wt.): 10% crumb rubber modifier, 90% bitumen 50/70 pen.

Table S4. Laboratory tests and equations used to calculate the different mechanical properties of the mixtures, according to standards

Properties	Laboratory test [test standard]	Equation		Test measurements and results [unit]
Thickness variation vs. compaction energy	Compactability<br>[EN 12697-10]	$\frac{1}{t(E)}=\frac{1}{t_\infty}-\left(\frac{1}{t_\infty}-\frac{1}{t_0}\right)\cdot\exp\left(\frac{-E}{T}\right)$	(Eq. 1)	($t(E)$) thickness of the compacted specimen as a function of the compaction energy [mm];<br>(t_∞) minimum possible thickness of the specimen [mm];<br>(t_0) initial thickness of the specimen [mm];<br>(E) compaction energy by impact, expressed considering 21 Nm/blow;<br>(T) resistance to compaction by impact [number of blows]
Stiffness	Dynamic stiffness modulus<br>[EN 12697-26]	$S_m=\frac{F\cdot(\mu+0.27)}{(z\cdot h)}$	(Eq. 2)	(S_m) Stiffness modulus measured by the test [MPa];<br>(F) maximum vertical load applied [N];<br>(z) horizontal strain amplitude during the loading cycle [mm];<br>(h) average specimen thickness [mm];<br>(μ) Poisson ratio [a constant value of 0.35 was assumed].
		$S'_m=S_m\cdot\{1-0.322\cdot[Log(S_m)-1.82]\cdot[0.6-k]\}$	(Eq. 3)	(S'_m) Stiffness modulus corrected for a load surface factor of 0.6 [MPa];<br>(k) load surface factor measured
Fatigue law under cyclic loading	Resistance to fatigue<br>[EN 12697-24]	$Log(\varepsilon_0)=a_0+a_1\cdot Log(N)$	(Eq. 4)	(ε_0) initial strain amplitude;<br>(N) load cycles until fatigue failure;<br>(a_0, a_1) material constants obtained from the 4PB-PR fatigue test
Ultrasonic elastic constants	Ultrasonic pulse velocity<br>[EN 12504-4]<br>[BS 1881: Part 203]<br>[ASTM D2845-00]	$G=D_b\cdot V_S^2$<br>$E=G\cdot\frac{(3\cdot V_P^2-4\cdot V_S^2)}{V_P^2-V_S^2}$<br>$\mu=\frac{(V_P^2-2\cdot V_S^2)}{2\cdot(V_P^2-V_S^2)}$	(Eq. 5)<br>(Eq. 6)<br>(Eq. 7)	(D_b) bulk density of the material [kg/m^3];<br>(V_S) shear-wave velocity [m/s];<br>(V_P) compression-wave velocity [m/s];<br>(G) shear modulus [Pa];<br>(E) Young´s modulus [Pa];<br>(μ) Poisson´s ratio

2. Figures

a) 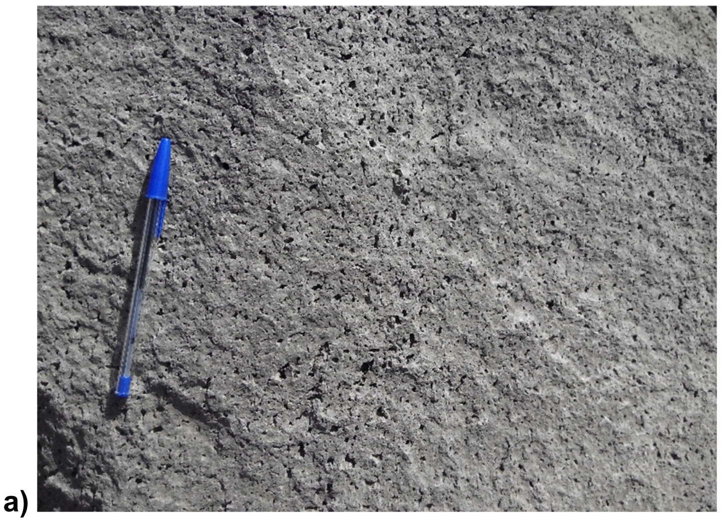 b) 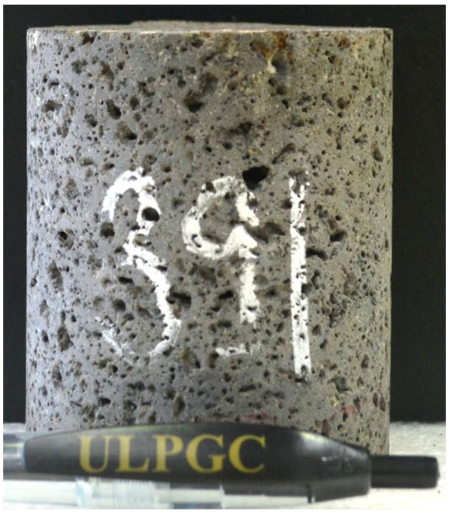

Fig. S1. Porous structure of the vesicular basalt (B-V): a) Detail of the structure of this common lithotype of volcanic rock; b) Core obtained from a fragment of B-V rock

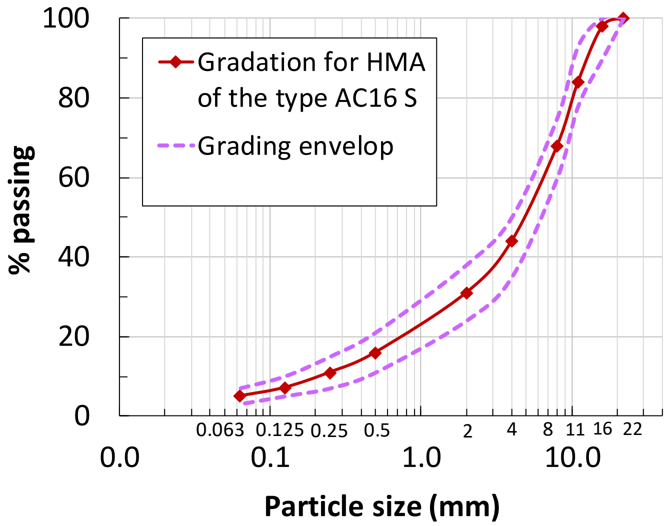

Fig. S2. Grading curve and limits of the grading envelope specified for semi-dense asphalt concrete (AC16 S)
